# West Nile Virus-Induced Cell Adhesion Molecules on Human Brain Microvascular Endothelial Cells Regulate Leukocyte Adhesion and Modulate Permeability of the *In Vitro* Blood-Brain Barrier Model

**DOI:** 10.1371/journal.pone.0102598

**Published:** 2014-07-18

**Authors:** Kelsey Roe, Beverly Orillo, Saguna Verma

**Affiliations:** Department of Tropical Medicine, Medical Microbiology and Pharmacology, Pacific Center for Emerging Infectious Diseases Research, John A Burns School of Medicine, University of Hawaii, Honolulu, Hawaii, United States of America; University of Kentucky Medical Center, United States of America

## Abstract

Characterizing the mechanisms by which West Nile virus (WNV) causes blood-brain barrier (BBB) disruption, leukocyte infiltration into the brain and neuroinflammation is important to understand the pathogenesis of WNV encephalitis. Here, we examined the role of endothelial cell adhesion molecules (CAMs) in mediating the adhesion and transendothelial migration of leukocytes across human brain microvascular endothelial cells (HBMVE). Infection with WNV (NY99 strain) significantly induced ICAM-1, VCAM-1, and E-selectin in human endothelial cells and infected mice brain, although the levels of their ligands on leukocytes (VLA-4, LFA-1and MAC-1) did not alter. The permeability of the *in vitro* BBB model increased dramatically following the transmigration of monocytes and lymphocytes across the models infected with WNV, which was reversed in the presence of a cocktail of blocking antibodies against ICAM-1, VCAM-1, and E-selectin. Further, WNV infection of HBMVE significantly increased leukocyte adhesion to the HBMVE monolayer and transmigration across the infected BBB model. The blockade of these CAMs reduced the adhesion and transmigration of leukocytes across the infected BBB model. Further, comparison of infection with highly neuroinvasive NY99 and non-lethal (Eg101) strain of WNV demonstrated similar level of virus replication and fold-increase of CAMs in HBMVE cells suggesting that the non-neuropathogenic response of Eg101 is not because of its inability to infect HBMVE cells. Collectively, these results suggest that increased expression of specific CAMs is a pathological event associated with WNV infection and may contribute to leukocyte infiltration and BBB disruption *in vivo*. Our data further implicate that strategies to block CAMs to reduce BBB disruption may limit neuroinflammation and virus-CNS entry via ‘Trojan horse’ route, and improve WNV disease outcome.

## Introduction

Since its introduction to the United States in 1999, West Nile virus (WNV), a mosquito-borne flavivirus classified as an NIAID Category B Priority Pathogen, has emerged as a leading cause of viral encephalitis, with more than 5,000 cases including nearly 250 deaths in 2012. WNV is an enveloped positive stranded RNA virus and is closely related to other human pathogens including dengue, yellow fever, Japanese encephalitis and tick-borne encephalitis viruses. Currently there are no therapeutic drugs or vaccines for WNV approved for human use [1]. The fatality rate is approximately 10% for hospitalized WNV cases and up to 70% of the survivors of WNV-encephalitis experience persistent neurological deficits for several months [2]. The pathogenesis of WNV in humans is not well characterized but WNV infection in mice mimics human WNV disease, thus making it a good model to understand the mechanisms that cause WNV disease. WNV infection triggers effective innate immune responses, which collectively mediate virus clearance from the periphery and control its dissemination in the brain, however in subset of patients WNV enters the central nervous system (CNS) [3]. Therefore, WNV neuropathogenesis is mainly dependent on the ability of the virus to enter the brain and replicate within resident cells including neurons and astrocytes [3]. Increased leukocyte infiltration, specifically CD8^+^ T cells are critical for clearing virus infection from the CNS, although migrating inflammatory monocytes and T cells also contribute to neuropathology by potentiating inflammation [4], [5]. Leukocytes entering the CNS must cross the blood-brain barrier (BBB) and one of the routes of WNV-CNS entry is also proposed to be via ‘Trojan horse’ mechanism by infected leukocytes [5].

The BBB is a selective cellular border made up of specialized cerebral microvascular endothelial cells that protects the CNS from blood-borne dangers. The BBB-endothelial cells interacts with perivascular structures like pericytes and astrocytes and have characteristic properties defined by high transendothelial electrical resistance, the expression of tight junction proteins (TJP) sealing the paracellular spaces, and a low pinocytotic activity [6], [7]. These features limit transcellular and paracellular movement of peripheral immune cells and molecules [8]. However, infection with neurotropic pathogens results in increased migration of leukocytes into the CNS, a key element of innate and adaptive immunity [9]. Leukocyte trafficking across the BBB, a very coordinated process including tethering, rolling and adhesion followed by transmigration, is governed by the interactions of endothelial cell adhesion molecules (CAMs) with their ligands, matrix metalloproteinases (MMPs) and chemokines [10], [11]. Endothelial CAMs such as immunoglobulin superfamily members (intracellular adhesion moleulce, ICAM-1; vascular cell adhesion protein, VCAM-1) and selectins interact with their leukocyte integrins counterparts (very late antigen 4, VLA-4; and lymphocyte function-associated antigen1, LFA-1) and, in concert with chemotactic chemokines, facilitate rolling and adhesion of leukocytes on the endothelial wall. Under healthy conditions the endothelial cells of the BBB express very low levels of CAMs, however the expression of multiple CAMs including ICAM-1, VCAM-1, and selectins upon inflammatory stimulation multiple sclerosis (MS) [12] or infection with viruses such as human immunodeficiency virus (HIV) [13], [14] and herpes simplex virus [15].

Our recent study demonstrated that the disruption of BBB in WNV-infected mice correlated with loss of TJPs and increased MMPs in the brain [16]. Using a human *in vitro* BBB model we have also shown that the transit of cell-free virus does not alter the permeability of the model [17]. In addition, we observed that WNV-induced expression of MMP-9 and -3 in human primary astrocytes, but not human brain microvascular endothelial (HBMVE) cells, is responsible for the degradation of TJP of HBMVE cells, suggesting that WNV-induced neuroinflammation may contribute to BBB disruption [18]. Infiltrating macrophages and T cells are critical for controlling infection and clearing WNV in the brain [3], [19]. Conversely, they are also proposed to be a route of virus-CNS entry and source of high levels of pro-inflammatory cytokines and chemokines in the brain [20], [21]. However, little is known about the underlying mechanisms of leukocyte transmigration and their role in BBB disruption associated with WNV infection. Therefore, the objective of the present study was to use transwell cultures of brain endothelial cells to examine the effect of leukocyte transmigration on the permeability of the *in vitro* BBB model and to further understand the role of WNV-induced CAMs in the transmigration of leukocytes across the BBB. Our results report CAMs such as ICAM-1, VCAM-1, and E-selectin are induced following WNV infection in human endothelial cells and mouse brain, blocking of which results in significant reduction of the adhesion of leukocytes to HBMVE cells and disruption of BBB model. We further compare the virus replication kinetics and induction of CAMs in HBMVE cells infected with neurovirulent NY99 and non-lethal Eg101 strain of WNV.

## Results

### The integrity of the *in vitro* BBB model is compromised following transmigration of monocytes

**Figure 1:**
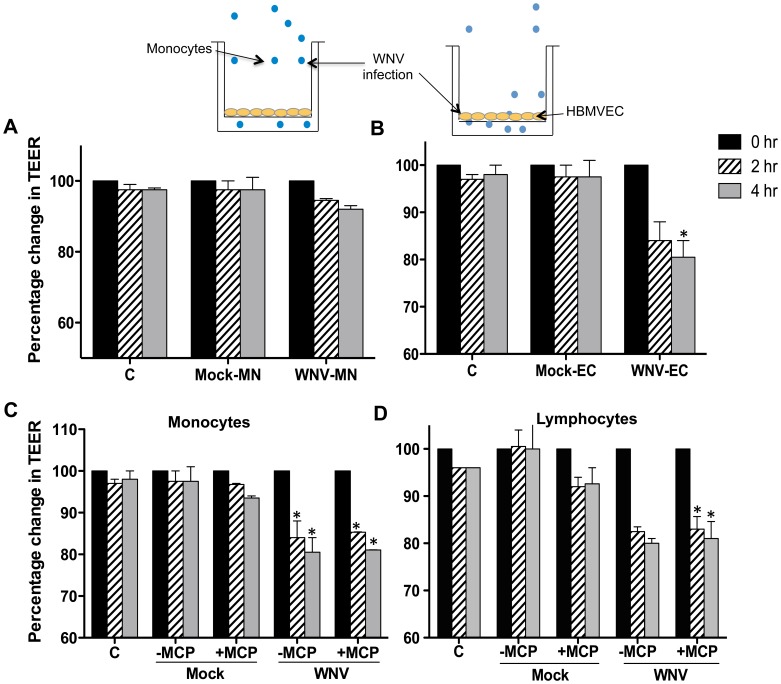
WNV infection of HMBVE cells, but not of leukocytes, mediates the disruption of the *in vitro* BBB model. The effect of leukocyte migration on the permeability of the *in vitro* BBB model was determined by TEER measurements. (**A)** WNV infected monocytes (2.5 x 10^5^) at 24 hrs after infection were added in the upper compartment of the uninfected BBB model and permeability was measured at 2 and 4 hrs after incubation. (**B**) At 72 hrs after WNV infection of BBB model, transmigration of uninfected monocytes significantly decreased the TEER as compared to control. (**C and D**) A chemotactic gradient was established by the addition of 100 ng/mL recombinant human CCL2 to the lower compartment and uninfected monocytes and lymphocytes were allowed to migrate across infected BBB. Results are the mean of data from two independent experiments in duplicate. *p<0.05 compared to mock. C, inserts with control HBMVE cells without leukocytes.

The acute infection of WNV *in vivo* is associated with the disruption of BBB [16]. Others and we have also shown that enhanced infiltration of leukocytes, both monocytes and T cells into the brain is one of the hallmarks of WNV-associated neuropathology in mice [16], [22]. Since leukocyte infiltration is shown to cause BBB disruption, in this study we first investigated how the migration of monocytes across the *in vitro* BBB model affects its integrity. To further delineate the role of WNV infected-leukocytes versus -endothelial cells in BBB disruption, we conducted parallel experiments using the transmigration of either WNV-infected monocytes at day 2 after infection across the uninfected BBB models (Fig. 1A) or uninfected monocytes across the WNV-infected BBB models at day 3 after infection (Fig. 1B). Incubation of the uninfected BBB model with infected monocytes (Fig. 1A) and lymphocytes (data not shown) did not result in a significant change in the transendothelial electrical resistance (TEER) following 2 and 4 hrs after transmigration. On the other hand, at 2 hrs after incubation of uninfected monocytes with WNV infected-inserts (day 3 after infection), there was a significant reduction in the percentage change in TEER as compared to the mock-infected controls, which further decreased at 4 hrs after incubation (Fig. 1B). Since the presence of chemokines such as monocyte chemotactic protein 1 (MCP-1 or CCL2) in the lower chamber is known to chemoattract leukocytes across the BBB, we next analyzed the influence of CCL2 on the permeability of the WNV-infected BBB model following incubation with uninfected monocytes and lymphocytes. As seen in Fig. 1C and D, after 2 and 4 hrs, incubation with both monocytes and lymphocytes reduced the TEER of the BBB model, however the presence of MCP-1 in the lower compartment of the transwell system did not further altered the TEER values. Together, these results suggest that WNV infection of leukocytes did not contribute significantly to the loss of resistance of the *in vitro* BBB model, rather it is the infection of BBB endothelial cells that mediated the disruption of the integrity of the BBB.

### WNV induces the expression of cell adhesion molecules in the HBMVE cells and mice brain

**Figure 2:**
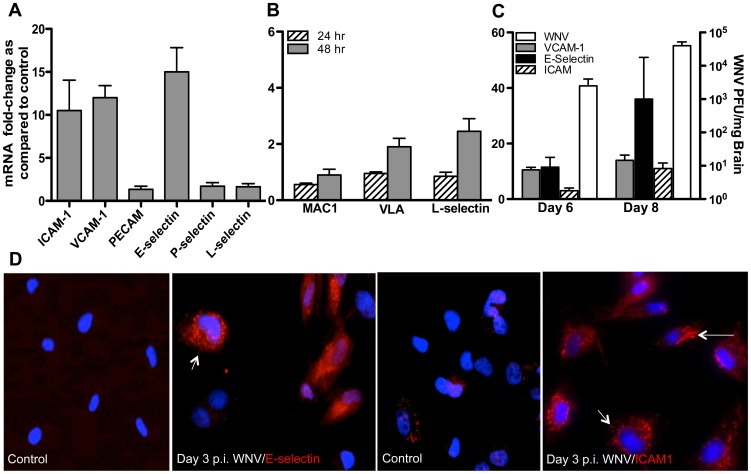
WNV induces expression of specific CAMs in the HBMVE cells and mouse brain. (**A**) The changes in the mRNA of multiple CAMs was measured in HBMVE cells at 72 hours after infection. (**B**) qRT-PCR was conducted on the RNA extracted from WNV-infected human monocytes to determine fold-change in the VLA-4, MAC-1 and L-selectin gene expression. (**C**) WNV titers and fold-change of ICAM-1, VCAM-1 and E-selectin in WNV-infected mouse brain as determined by qRT-PCR. The data are normalized to the values of GAPDH and are expressed as relative fold increase compared to uninfected controls. (**D**) Validation of the increase in the protein expression of ICAM-1 and E-selectin in HBMVE cells at 72 hrs after infection by immunostaining. The data are expressed as means ± SD from two independent experiments in duplicate. *p<0.05 compared to mock.

The CAMs bind to their ligands on leukocytes to facilitate their entry into the CNS. Previous work in our lab has demonstrated that WNV infection increased the mRNA expression of ICAM, VCAM and E-selectin in HBMVE cells. To further determine the specificity of these CAM's, in this study we looked at the mRNA expression of other important CAMs. Our results showed that the mRNA expression of P-selectin, L-selectin and PECAM did not change at 72 hrs after WNV infection of HBMVE cells (Fig. 2A), while increase in ICAM, VCAM and E-selectin correlated with peak virus titers [17]. In addition, we also analyzed the expression of key leukocyte adhesion molecules in WNV-infected monocytes, which may act as ligands for endothelial cell CAMs. As seen in Fig. 2B, while the mRNA expression of macrophage antigen 1 (MAC-1), VLA-4 and L-selectin did not change at 24 hrs, there was a modest increase in the expression of L-selectin at 48 hrs after infection. We next confirmed the increased protein expression of ICAM-1 and E-selectin in HBMVE cells by immunostaining. As demonstrated in Fig. 2D, whilst the mock-infected HBMVE cells did not exhibit any staining of E-selectin and ICAM-1, a very strong signal was observed in WNV-infected HBMVE at day 3 after infection. To further validate the ability of WNV to increase the expression of these CAMs *in vivo*, mRNA expression of ICAM-1, VCAM-1 and E-selectin was analyzed using qRT-PCR in the brains of WNV-infected mice (100 PFU via footpad route). As compared to the mock-infected controls, the mRNA transcripts of ICAM-1, VCAM-1 and E-selectin in the brain did not change at day 4 after infection (data not shown), however a 2- to 9-fold increase was observed at day 6, which further increased at day 8 after infection (Fig. 2C). The increased expression of these CAMs correlated very well with the peak WNV titers at day 8 after infection (Fig. 2C) and other markers of WNV-neuropathogenesis such as disruption of the BBB and infiltration of leukocytes as demonstrated in our previous studies [16]. Similar increase in the mRNA expression of these CAMs (20- to 42-fold) was also observed in the brains of mice infected via intracranial route at day 6 after infection when the virus titers were at peak (data not shown), thus confirming that the induction of these CAMs is linked to robust virus replication in the brain.

### WNV NY99 and Eg101 infection results in similar levels of virus replication and induction of CAMs

**Figure 3:**
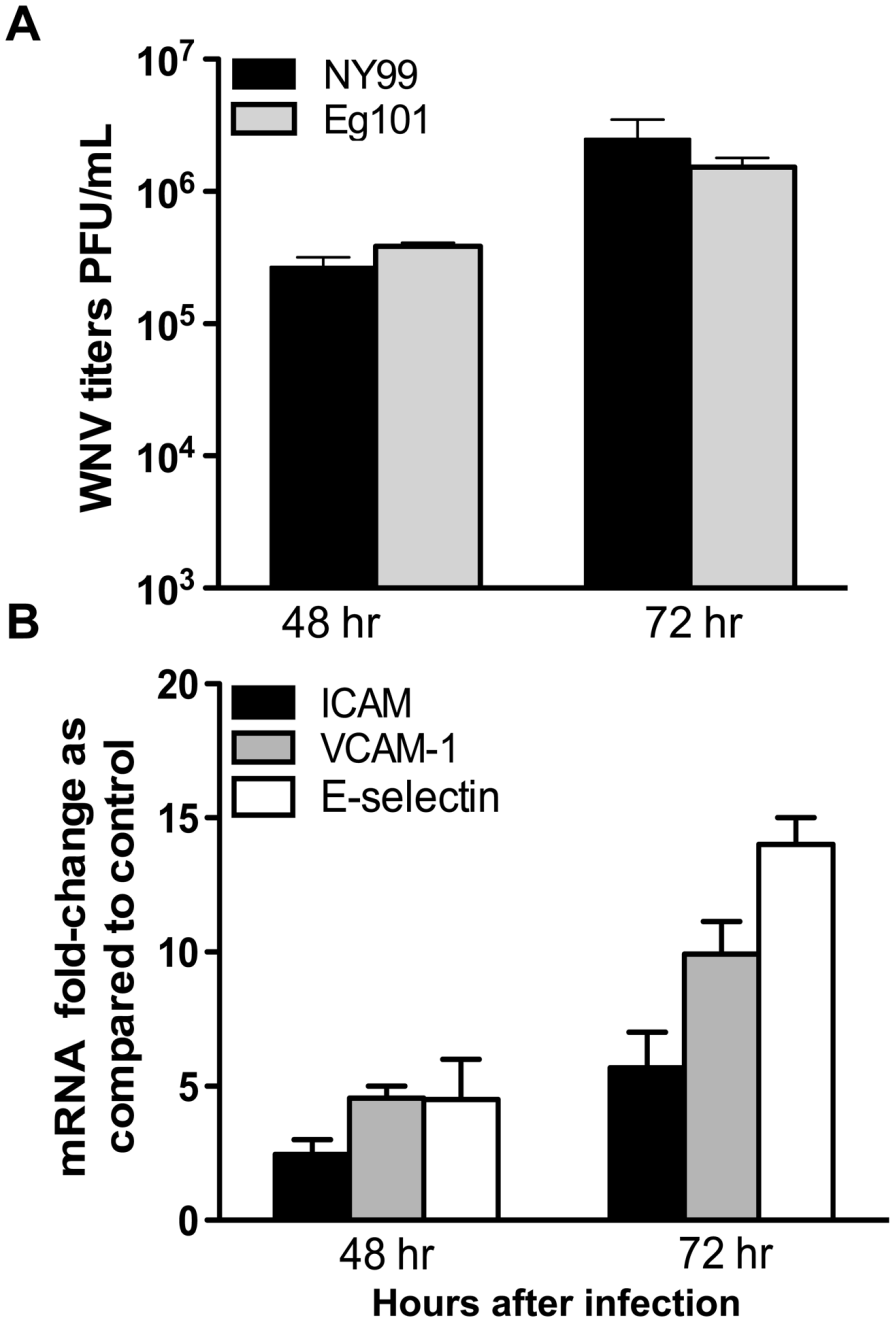
WNV-Eg101 efficiently infects HBMVE cells and induces CAMs. Primary human HBMVE cells were infected with WNV-NY99 or Eg101 (MOI5). (**A**) Culture supernatants recovered at 48 and 72 hrs after infection were used to determine titers using plaque assay on Vero cells. Data represent the mean ± SD PFU per mL of supernatant from two independent experiments. (**B**) qRT-PCR analysis of CAMs in Eg101 infected HBMVE cells at 48 and 72 hrs after infection. The data are normalized to the values of GAPDH and are expressed as relative fold increase compared to uninfected controls.

The neuroinvasive potential of WNV is strain dependent. While NY99 strain is highly neurovirulent, non-lethal Eg101 is not considered to be neuroinvasive. Therefore, to further understand the differential response of NY99 and Eg101 strain in the BBB-endothelial cells, we first compared the replication kinetics of these strains in HBMVE cells. As seen in Fig. 3A, NY99 and Eg101 strain replicated to similar levels in HBMVE cells at 48 and 72 hrs after infection, and did not exhibit any cytopathic effect (data not shown). Analysis of CAMs response to Eg101 demonstrated that mRNA of all three CAMs, ICAM-1, VCAM-1 and E-selectin were induced in infected HBMVE cells at 48 hrs time point, which further increased at 72 hrs after infection (Fig. 3B). This increase was comparable to the CAM induction observed in NY99-infected HBMVE cells (Fig. 2A) suggesting that in an *in vitro* culture system, when infected with the same MOI, the response of HBMVE cells culture system to both these strains of WNV is similar at the level of virus replication and induction of CAMs. Based on these results, NY99 strain was used to delineate the role of CAMs in WNV-associated BBB disruption.

### Blocking of CAM's limits leukocyte adhesion to the HBMVE cells

**Figure 4:**
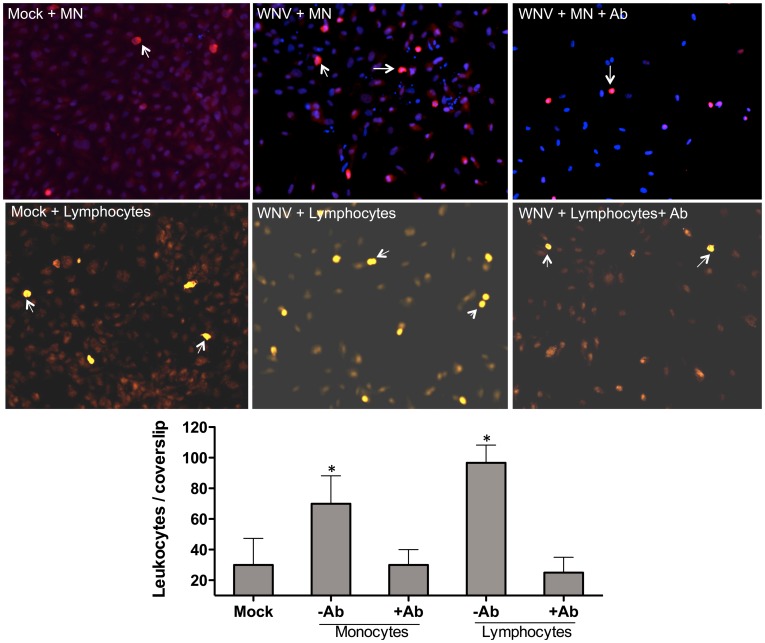
Blocking of cell adhesion molecules limits leukocyte adhesion to the HBMVE cells. HBMVE cell monolayers on the coverslips were infected with WNV for 72^5^ monocytes or lymphocytes were added to the cells in the presence or absence of the cocktail of neutralizing antibodies against CAMs (ICAM-1, VCAM-1 and E-slectin). After two hrs, the non-adhered leukocytes were vigorously washed off and adherent cells were stained with CD45 (white arrows). (**A**) Immunofluorescence micrograph of CD45-stained monocytes and lymphocytes. (**B**) Quantitative representation of CD45^+^ cells from six different areas per coverslip from three independent experiments. *p<0.05 compared to mock.

The interactions of leukocytes with the CAM's on the brain endothelial cells play an important role in their CNS transmigration. Since our results demonstrated increase of three specific CAMs (Fig. 2, ICAM-1 VCAM-1 and E-selectin), we next analyzed the role of these CAMs in mediating the adhesion of leukocytes to the HBMVE cells. As seen in Fig. 4, under mock-infected conditions only a small number of monocytes adhered to the HBMVE cells, which increased significantly to the WNV-infected HBMVE cells (day 3 after infection, p<0.05). However, pretreatment of HBMVE cells with a cocktail of blocking antibodies against ICAM-1, VCAM-1 and E-selectin before the co-culture dramatically reduced the number of monocytes adhered to the infected HBMVE cells (Fig. 4). We also observed similar results of increased attachment of lymphocytes to the WNV-infected HBMVE cells, which was significantly reduced in the presence of blocking antibodies to CAMs.

### Blocking antibodies against CAMs can limit disruption of the *in vitro* BBB model and leukocyte transmigration

**Figure 5:**
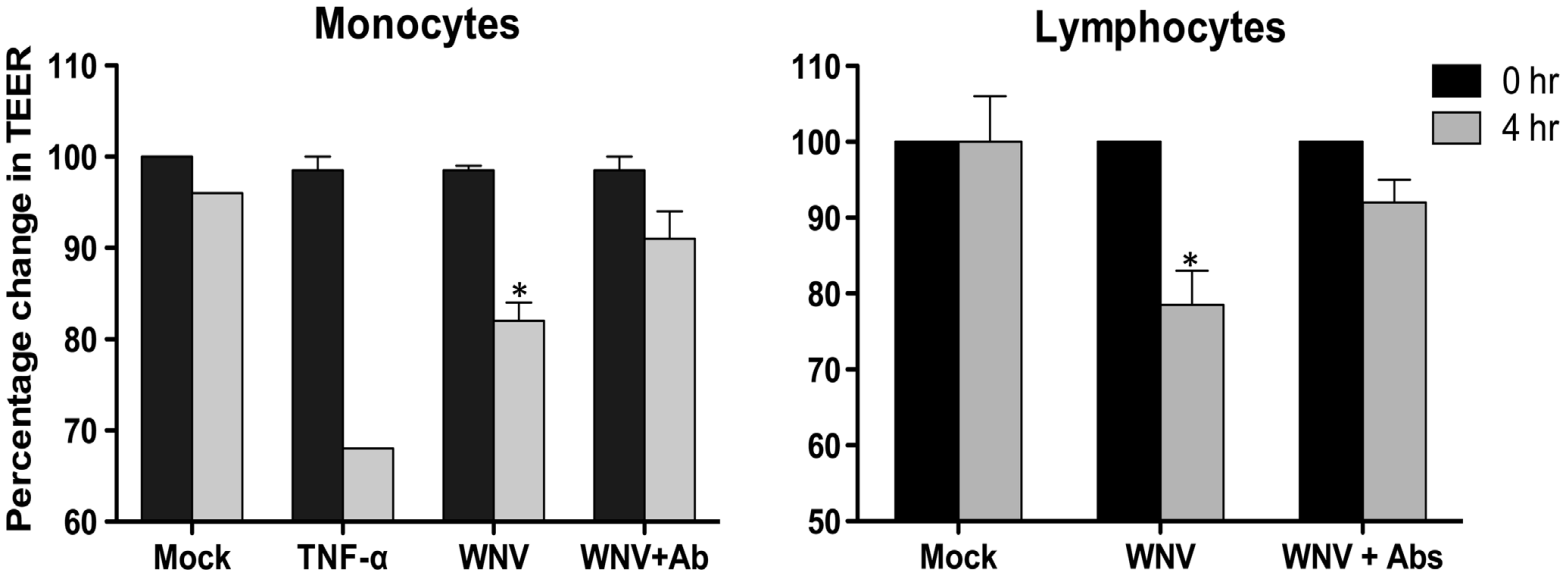
Neutralizing antibodies against cell adhesion molecules partially reverse the disruption of the *in vitro* BBB model during leukocyte transmigration. The integrity of the *in vitro* BBB model was determined by measuring the TEER before and after four hrs of incubation with monocytes and lymphocytes in the presence or absence of the cocktail of neutralizing antibodies against CAMs. The decrease in the TEER values was represented as percentage change as compared to mock-infected controls. The data is mean ± SD of two independent experiments. *p<0.05 compared to mock.

After demonstrating the role of CAMs in leukocyte-HBMVE cell adhesion, we subsequently analyzed their role in the disruption of *in vitro* BBB model during the transmigration. As demonstrated in Fig. 5, incubation of the WNV-infected inserts with monocytes in the presence of a cocktail of neutralizing antibodies against CAMs (ICAM-1, VCAM-1 and E-selectin) reversed the decrease in the TEER values. A similar trend was observed during the incubation of lymphocytes, which showed a significant reduction in the percentage change in the TEER values in the presence of the blocking antibodies (Fig. 5).

**Figure 6:**
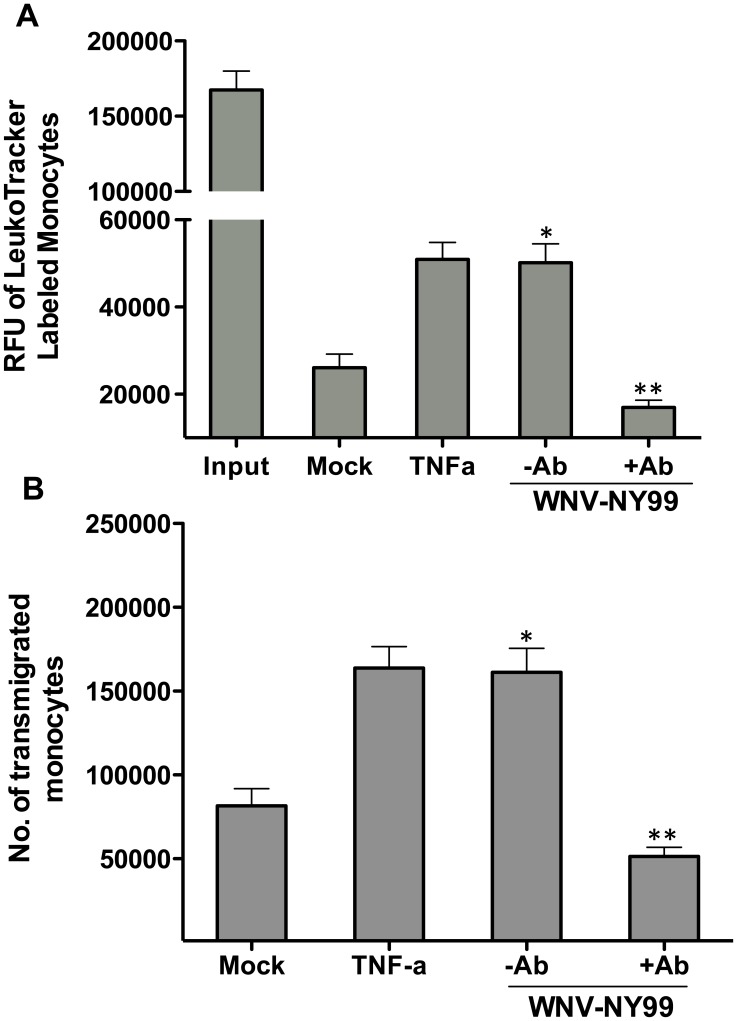
Blocking CAMs limits the transmigration of monocytes across the *in vitro* BBB model. Human monocytes stained with leukotracker dye were added to the upper compartment of the *in vitro* BBB model infected with NY99 strain of WNV in the presence or absence of neutralizing antibodies against ICAM-1, VCAM-1 and E-slectin. Treatment with TNF-α (0.125 ng/mL) was used as a positive control. After four hrs of incubation, the media of the lower compartment was collected and the flourescence was measured. (**A**) Flourescence absorbance (RFU) of the transmigrated monocytes in the lower compartment. (**B**) Absolute number of transmigrated monocytes extrapolated based on the standard curve generated using RFU of known number of leukotracker stained monocytes. The data is representative of two independent experiments. *p<0.05 compared to mock and **p<0.05 compared to WNV-infected.

Next, we determined whether blocking leukocytes adhesion to the endothelial cells using antibodies against CAMs also interferes with the leukocyte transmigration across the BBB model. Using fluorescent-tagged monocytes, we observed that the transendothelial migration of monocytes across the uninfected BBB model was minimal after 2 hrs of incubation (Fig. 6). Pretreatment of the *in vitro* BBB model with tumor necrosis factor-α (TNF-α) increased the number of migrated monocytes by >2 fold as represented by the increased fluorescence of the leukoTracker dye in the lower chamber (Fig. 6). Similarly, compared to the controls, WNV infection of the BBB model increased the number of monocytes crossing the BBB model by 2.5-fold. As seen in Fig. 6B, only 10–15% of the cells transmigrated across the uninfected BBB model (500,000 cells in upper compartment), which increased significantly to ∼35% (p<0.05) in the case of infected BBB model. However, as expected, in the presence of the blocking antibodies, there was a significant decrease in the transmigration of monocytes across the infected model and represented only 10% of the total monocytes in the upper chamber (p<0.05). Presence of blocking antibodies had no effect on the minimal monocytes migration across the uninfected models (data not shown). Collectively, our results indicate that infection of HBMVE cells with WNV increased the adhesion and migratory capacity of leukocytes resulting in the disruption of the BBB model.

## Discussion

Infection with WNV in the mouse model of WNV-encephalitis is characterized by BBB disruption and massive leukocyte infiltration into the brain. The actual basis of BBB disruption, however, has not been elucidated. We previously demonstrated that WNV infection in the HBMVE cells can be one of the routes of cell-free virus entry into the brain, however it does not compromise the integrity of the BBB model, suggesting that events other than direct virus infection are responsible for the BBB disruption. Here, we demonstrate that (i) transmigration of leukocytesacross the WNV-infected BBB model compromises its integrity, (ii) WNV-induced ICAM-1, VCAM-1 and E-selectin are critical in mediating the adherence of leukocytes to the HBMVE cells thereby facilitating their transmigration across the *in vitro* BBB model and (iii) blocking these CAMs significantly reduce disruption of the BBB model.

Important observation of our data was that the infection of leukocytes alone was not enough to cause the disruption of the BBB model. It was the infection and subsequent activation of HBMVE cells, specifically the up regulation of these CAMs that mediated the disruption of the BBB (Fig. 1 and 2). Additionally, our data indicate that WNV does not significantly modulate expression of VLA-4 and MAC-1 in leukocytes and this indirectly supports the notion that it is primarily the virus-induced activation of the BBB endothelium that is responsible for BBB disruption. In previous studies, avirulent strain of WNV (Sarafend) has been shown to induce expression of ICAM-1, VCAM-1 and E-selectin in human endothelial cells [23], although its function in mediating leukocyte infiltration was not described. In human monocytes, this is the first report showing that WNV (NY99)-induced changes in the expression of VLA-4 and MAC-1 is not as dramatic as CAMs induced in HBMVE cells. Our results using human cells partially agree with the recent mouse study demonstrating a modest increase in the VLA-4 expression in the subset of inflammatory macrophages (Ly6C+) recovered from the WNV-infected mice brain, although this increase was not significant [24]. Further, although the infectious dose and the route of infection were different, our results in mouse brain (Fig. 2C) are in accord with Dai *et al.*, who showed increased ICAM-1 mRNA expression in the mice infected with 1000 PFU of WNV 2741 isolate via intraperitoneal route [25].

*In vivo*, WNV replication is typically established in the brain of mice infected via footpad route by day 6 after infection and virus titers and WNV- induced inflammation peak by day 8 after infection [3], [16], [26]–[28]. Our *in vivo* data demonstrating significant increase of ICAM-1, VCAM-1 and E-selectin in the brain at day 8 after infection correlates with the peak of inflammatory cytokines levels and suggest that this may be one of the downstream responses of virus-induced inflammation in the brain. Based on our in vitro data, we speculate that increased CAMs in the brain is the response of BBB-endothelial cells to WNV infection in the CNS, however likelihood of other CNS cells in contributing to CAMs increase cannot be ruled out. Robust induction of cytokines such as TNF-α and interleukin-1β (IL-1β) is shown in WNV infected brain [26], [28]–[30] and it is highly likely that these inflammatory cytokines may be responsible for the induction of these CAMs as has been shown in other neuroinflammatory disorders [11], [31].

In order to determine the role of chemotactic gradient in leukocyte migration, we also assessed the effect of leukocyte-HBMVE interaction in the presence of CCL2. CCL2 has been shown to facilitate the chemotaxis of both monocytes and T cells *in vitro* and has been implicated in the pathogenesis of several virus diseases including HIV, dengue and WNV [4], [32]. CCl2 is highly expressed in dengue hemorrhagic fever/dengue shock syndrome patients and is proposed to contribute to vascular permeability changes, possibly by weakening tight junctions of vascular endothelium cells [33]. Similarly, presence of CCL2 in the lower chamber of the *Toxoplasma gondii*-induced *in vitro* BBB disruption model, further increased the transmigration of infected lymphocytes [34]. However, our results (Fig. 1) indicate that in our system, transmigration of monocytes and T cells in the presence of CCL2 did not further alter the permeability of the infected BBB model. However, this does not rule the possibility of other chemokines such as the CCL5 in chemoattracting leukocytes to the lower chamber of the model. Further, our data clearly demonstrate that the negligible leukocyte adhesion to uninfected HBMVE did not appear to be dependent upon the interaction with CAMs since blocking antibodies did not further decrease adhesion in the mock-infected controls. On the other hand, increased leukocyte adhesion to the infected HBMVE can be attributed to the increased expression of CAM's based on the observation that blocking antibodies markedly reduced the number of monocytes and lymphocytes adhered to the HBMVE. Our results are similar to other studies using Theiler's murine encephalitis virus (TMEV) demonstrating reversal in the leukocyte adhesion and transmigration in the presence of VCAM-1 in infected endothelial cells [35].

The infiltration of the leukocytes can have multiple downstream effects in WNV pathogenesis. First, WNV might promote its entry into the CNS through the BBB in a ‘Trojan horse’ manner, where infected monocytes and T cells gain entry into the CNS and disseminate virus to the neighboring brain cells. Such phenomenon has already been proposed and demonstrated in infection with several neurotropic pathogens including HIV, Venezuelan equine encephalitis virus (VEEV) and *T.gondii* [5], [34], [36], [37]. Second, recruited immune cells might contribute to immunopathology. Though leukocyte infiltration is critical to clear WNV from the brain, they may also be one of the causes of massive inflammation in the CNS leading to neuronal death via apoptosis. WNV-infected and activated monocytes and T cells have been shown to produce inflammatory cytokines and chemotactic chemokines [27], [38]. Recently, production of nitric oxide (NO) by WNV-infected macrophages in the brain has been associated with the pathogenic function of leukocytes [24]. Lastly, uncontrolled leukocyte transmigration can be one of the causes of the WNV-associated BBB disruption observed *in vivo* [16], [27]. Our current findings strongly suggest important role of specific WNV-induced CAMs in modulating the extent of transmigration of peripheral leukocytes into the brain, thereby causing BBB disruption.

In this study, we used a cocktail of blocking antibodies against all three CAM's, VCAM-1, ICAM and E-selectin instead of blocking each of these CAM's individually to address their independent roles and relative contribution in BBB disruption. We considered this approach based on the fact that all of these WNV-induced CAM's are critical in leukocyte trafficking and blocking one of them would not significantly affect different events underlying leukocyte transmigration. Consistent with this fact, it is shown that combinational treatment with anti-MAdCAM-1, VCAM-1 and ICAM-1 monoclonal antibodies led to more rapid remission in the experimental autoimmune encephalitis (EAE) model of MS than that obtained with individual antibodies alone [39]. Similarly, Steiner *et al.*, found that ICAM-1 and VCAM-1 have redundant roles in mediating shear resistant arrest of encephalitogenic T cells to the BBB endothelial cells and only in the functional absence of both was the complete abrogation of T cell arrest on the BBB observed [40].

Increasing evidence suggests that the ability of the WNV to invade CNS is strain specific [41]. The non-neurovirulent nature of Eg101 is attributed to several factors including differences in innate immune response [42] and not able to cross the BBB, however its interaction with BBB cells is not yet characterized. Human brain endothelial cell infection with Eg101 is not reported so far and our data for the first time indicate that the replication kinetics of Eg101 and Ny99 strain in HBMVE cells was comparable (Fig 3A). This observation is indirectly supported by similar studies demonstrating that another non-pathogenic WNV strain, MAD78 can replicate as efficiently as NY strain in BBB-endothelial cells [43], [44]. On the other hand, Hasebe and group demonstrated that virus like particles (VLP) of NY99 transported efficiently from the apical to basolateral side of human endothelial cells, whereas Eg101-VLPs hardly transported [45]. Further *in vivo* studies are warranted to delineate differences between NY99 and Eg101 at the level of CNS entry, BBB disruption and leukocyte infiltration. However, our results imply that the difference in the neuroinvasive ability between Eg101 and NY99 is not at the level of virus replication and subsequent induction of CAMs in HBMVE cells. It is likely that Eg101 is less neuroinvasive because the host immune response is able to clear Eg101 more efficiently in the periphery as compared to NY99, which may lead to lower viremia resulting in reduced CNS entry of Eg101.

Targeting CAMs either directly using neutralizing antibodies or by blocking upstream signaling in animal models is being proposed as an attractive and feasible strategy for therapeutic intervention of reducing transendothelial migration of leukocytes and neuroinflammation in inflammatory diseases such as asthma and polymicrobial sepsis [35], [46]. Giri *et al.* showed that β-amyloid-mediated migration of monocytes across BBB model was inhibited by blocking CAMs [47]. The data is limited in virus infections, although it has been shown that Anandamide inhibits Theiler's virus induced VCAM-1 in brain endothelial cells resulting in reduced leukocyte transmigration across the BBB model [35]. Further, WNV studies using ICAM-1-/- mice provide direct evidence of the pathogenic role of ICAM-1 by demonstrating decreased BBB disruption along with reduced leukocyte infiltration and inflammation in infected ICAM-/- brains [25]. Further, its has been recently demonstrated that treatment with VLA-4 antibody can reduce CNS infiltration of macrophages and increase survival of mice infected with the Sarafend strain of WNV via intranasal route following [24]. These studies collectively suggest the possibility of therapeutic effects of temporarily targeting specific CAMs to improve pathogenesis of WNV and other encephalitis causing viruses.

In summary, our results provide insights into the mechanisms by which WNV modulates brain endothelium to facilitate leukocyte trafficking into the CNS. Increased expression of specific CAMs may be a pathological event associated with WNV infection and may contribute to BBB disruption *in vivo*. Collectively, our data implicate that strategies to block CAMs to keep leukocyte infiltration and BBB disruption in check will limit neuroinflammation and virus-CNS entry via ‘Trojan horse’ route, and improve WNV disease outcome.

## Materials and Methods

### Ethics statement

This study was specifically approved by the University of Hawaii Institutional Animal Care and Use Committee (IACUC, protocol number 08-265), and conducted in strict accordance with guidelines established by the National Institutes of Health and the University of Hawaii IACUC. All animal experiments were conducted in consultation with veterinary and animal care staff at the University of Hawaii in animal biosafety level-3 laboratory. Mice that exhibited severe disease were immediately euthanized using CO_2_ to limit suffering.

### Cells and Virus

Low-passage primary HBMVE cells were purchased from Cell Systems Corporation (Cell Systems Corp., Kirkland, WA) and propagated on attachment factor coated flasks with CSC-Complete medium (Cell Systems Corp.) as described previously [17]. All experiments were performed with cells between passages 6 to 10. A stock of lineage I WNV strain NY99, originally isolated from a crow brain in New York and propagated in Vero cells, was used for all infection experiments at MOI 5 for HBMVE cells and MOI 1 for monocytes and lymphocytes. WNV Eg101, a gift from Dr. Duane Gubler, was used for infections at the MOI 5 in some experiments. HBMVE cells were infected with WNV as described previously [17], [48] and cells and supernatant were harvested at various time points after infection.

### PBMC isolation and separation

An Institutional Review Board-approved protocol was used for the isolation of PBMCs from 60–80 mL anti-coagulated blood of healthy donors. Monocytes were separated using the EasyStep Negative Selection Human Monocyte Enrichment Kit (Cat#19059, StemCell Technologies) following the manufacturers instructions. Monocytes-depleted leukocytes (lymphocytes) were separated by allowing the monocytes to adhere onto tissue culture plates for at least 2 hrs in a 37°C incubator.

### *In vitro* BBB model, WNV infection and permeability assay

*In vitro* monolayer BBB models were constructed by plating 5 x 10^4^ HBMVE cells on a BioCoat Cell Environment Human Fibronectin PET insert with 3.0 µm pores in a 24-well plate (BD Bioscience, Bedford, MA) as described previously [17], [18]. The integrity of the *in vitro* BBB model was determined every day from day 3 after seeding by measuring the TEER EMVOX and was expressed as Ω/cm^2^. At day 8–10 after seeding, the *in vitro* BBB models were infected with WNV at MOI 5. At day 3 after infection, the inserts were used for transmigration and permeability assays in the presence or absence of a cocktail of neutralizing antibodies against CAMs (ICAM-1, 10 ug/mL; VCAM, 20 ug/mL; and E-selectin, 10 ug/mL from R&D systems). When used at these concentrations, all of these antibodies are reported to have the specificity of inhibiting adhesion of monocytic U937 cells by 80–100% (R&D systems) and have been used in several adhesion studies [49]. A total of 2.5 x 10^5^ monocytes or lymphocytes in 400 µL were added to the upper chambers of the *in vitro* BBB models and incubated with or without the presence of the cocktail of neutralizing antibodies. In selected experiments, 100 ng/mL MCP-1 was added in the lower chamber medium as a chemoattractant. After 2 and 4 hrs of incubation, the BBB integrity was measured by TEER assay as described previously [17], [18]. For positive control, the inserts were treated with 0.125 ng/mL of TNF-α.

### Adhesion assay

Confluent monolayers of HBMVE cells grown on coverslips in 24 well plates were infected with WNV at MOI 5. At day three of infection, the coverslips were incubated with the cocktail of neutralizing antibodies against ICAM-1, VCAM-1 and E-selectin (5 ug/mL each) for one hour prior to the adhesion assay. 2.5 x 10^5^ monocytes or lymphocytes in 0.5 mL media were then added and incubated with HBMVE in the presence or absence of the blocking antibodies for one hour at 37°C as described previously [49]. At the end of the incubation period, the supernatant with the non-adherent leukocytes (monocytes or lymphocytes) was removed and the HBMVE monolayers were vigorously washed four times to remove loosely attached cells. The HBMVE monolayers with adherent leukocytes were fixed in 4% paraformaldehyde (PFA) for 10 minutes and were stained with DAPI for nucleus marker and FITC conjugated anti-CD45 (a leukocyte marker) diluted 1∶100 and examined using a Zeiss microscope as described previously [18]. Controls included uninfected HBMVE cells co-cultured with leukocytes with and without neutralizing antibodies. The number of leukocytes bound to the monolayers was determined by counting the number of adherent monocytes and lymphocytes in two central and four peripheral randomly selected fields of 3 coverslips from two independent infections.

### Transmigration assay

For the transmigration assays, freshly isolated monocytes and lymphocytes were first tagged by staining with Leukotracker dye as per the instructional manual (Cell Biolabs). Confluent transwell inserts with a mean TEER >110 Ω were carefully transferred to fresh wells containing 500 µL of the medium and 5 x 10^5^ monocytes and lymphocytes in 500 µL of buffer were added to the inserts and allowed to migrate at 37°C. After 4 hrs, the medium from the lower chamber was collected and the fluorescence of Leukotracker dye was quantitated at 480/520 nm using a Victor3 microtiter reader.

### Analysis of cell adhesion molecules in WNV-infected human endothelial cells, monocytes and mice brain

In the HBMVE monolayer, mRNA expression of multiple CAMs at 72 hrs after infection was determined using microarrays and increased expression of VCAM-1, ICAM-1 and E-selectin was validated by qRT-PCR [17]. The RNA extracted from monocytes after 48 hrs of WNV infection was used to measure the mRNA expression of VLA-4, MAC-1 and L-selectin by qRT-PCR using specific primers (Table 1). For protein expression of ICAM-1 and E-selectin at 72 hrs, mock- and WNV-infected HBMVE cells were fixed in 4% PFA and immunostained using anti-ICAM-1, or monoclonal mouse anti-E-selectin followed by streptavidin 596 conjugated anti-mouse secondary antibodies as described previously [17]. Eight- to 10-week old C57BL/6 mice were infected with 100 PFU WNV (NY99 strain) via the footpad as described previously in accordance with the University of Hawaii at Manoa animal studies guidelines [16], [28]. Brains harvested at different time points after infection were used for RNA extraction and qRT-PCR analysis using specific primers as described in Table 1. The mRNA levels were normalized against GAPDH and expressed as fold increase over RNA levels in mock-infected brain tissues [28].

**Table 1:** Primer sequences used for qRT-PCR.

Gene (Accession No.)	Primer Sequence (5'-3')	Amplicon	
		(bp)	Tm (°C)
Mac-1 (J03925)-Human			
Forward	AACTGTGATGGAGCAATT	104	58
Reverse	GGTTGTTCTGGAACTCTT		
L-selectin (NM_000655)-Human			
Forward	ATTTCCTGGCACATCATG	95	59
Reverse	ATTGTCTCGGCAGAATCT		
LFA (NM_002209)-Human			
Forward	TGACCAGAACACCTATCT	136	58
Reverse	CGAACCATCAAACAGAAAT		
ICAM-1 (NM_010493)-Mice			
Forward	ATAACTGGACTATAATCATTCTG	119	57
Reverse	AGCCTTCTGTAACTTGTAT		
E-selectin (M87862)-Mice			
Forward	CATGACGTATGATGAAGC	98	57
Reverse	GATTGGAGTTAAGGTAGTTG		
VCAM-1 (NM_011693)-Mice			
Forward	CTCTAGCAAGACCCTTTA	149	57
Reverse	CATCTTCACAGGCATTTC		

### Statistical analysis

All mRNA quantitation data are reported as mean ± standard error of at least two independent experiments in duplicate. Unpaired student t-test was used to compare the values of leukocyte adhesion and transmigration assays and two-way analysis of variance (ANOVA) with Bonferonni post-tests was used to compare the values of BBB permeability assays using GraphPad Prism 5.0 (GraphPad software, San Diego, CA). *p*<0.05 was considered as statistically significant for all analyses.

## References

[1] Diamond MS (2009) Progress on the development of therapeutics against West Nile virus. Antiviral Res 83: 214–227. 19501622 10.1016/j.antiviral.2009.05.006 PMC2759769

[2] Sejvar JJ, Haddad MB, Tierney BC, Campbell GL, Marfin AA, et al (2003) Neurologic manifestations and outcome of West Nile virus infection. JAMA : the journal of the American Medical Association 290: 511–515. 12876094 10.1001/jama.290.4.511

[3] Suthar MS, Diamond MS, Gale M Jr (2013) West Nile virus infection and immunity. Nat Rev Microbiol 11: 115–128. 23321534 10.1038/nrmicro2950

[4] Getts DR, Terry RL, Getts MT, Muller M, Rana S, et al (2008) Ly6c+ "inflammatory monocytes" are microglial precursors recruited in a pathogenic manner in West Nile virus encephalitis. J Exp Med 205: 2319–2337. 18779347 10.1084/jem.20080421 PMC2556789

[5] Wang S, Welte T, McGargill M, Town T, Thompson J, et al (2008) Drak2 contributes to West Nile virus entry into the brain and lethal encephalitis. J Immunol 181: 2084–2091. 18641347 10.4049/jimmunol.181.3.2084 PMC2494872

[6] Persidsky Y, Ramirez SH, Haorah J, Kanmogne GD (2006) Blood-brain barrier: structural components and function under physiologic and pathologic conditions. J Neuroimmune Pharmacol 1: 223–236. 18040800 10.1007/s11481-006-9025-3

[7] Abbott NJ (2005) Dynamics of CNS barriers: evolution, differentiation, and modulation. Cell Mol Neurobiol 25: 5–23. 15962506 10.1007/s10571-004-1374-y PMC11529509

[8] Deli MA, Abraham CS, Kataoka Y, Niwa M (2005) Permeability studies on in vitro blood-brain barrier models: physiology, pathology, and pharmacology. Cell Mol Neurobiol 25: 59–127. 15962509 10.1007/s10571-004-1377-8 PMC11529645

[9] Kanmogne GD, Schall K, Leibhart J, Knipe B, Gendelman HE, et al (2007) HIV-1 gp120 compromises blood-brain barrier integrity and enhances monocyte migration across blood-brain barrier: implication for viral neuropathogenesis. J Cereb Blood Flow Metab 27: 123–134. 16685256 10.1038/sj.jcbfm.9600330 PMC2232899

[10] Dietrich JB (2002) The adhesion molecule ICAM-1 and its regulation in relation with the blood-brain barrier. J Neuroimmunol 128: 58–68. 12098511 10.1016/s0165-5728(02)00114-5

[11] Stanimirovic D, Satoh K (2000) Inflammatory mediators of cerebral endothelium: a role in ischemic brain inflammation. Brain Pathol 10: 113–126. 10668901 10.1111/j.1750-3639.2000.tb00248.x PMC8098501

[12] Avolio C, Giuliani F, Liuzzi GM, Ruggieri M, Paolicelli D, et al (2003) Adhesion molecules and matrix metalloproteinases in Multiple Sclerosis: effects induced by Interferon-beta. Brain Res Bull 61: 357–364. 12909305 10.1016/s0361-9230(03)00098-4

[13] Nottet HS, Persidsky Y, Sasseville VG, Nukuna AN, Bock P, et al (1996) Mechanisms for the transendothelial migration of HIV-1-infected monocytes into brain. J Immunol 156: 1284–1295. 8558009

[14] Eugenin EA, Gamss R, Buckner C, Buono D, Klein RS, et al (2006) Shedding of PECAM-1 during HIV infection: a potential role for soluble PECAM-1 in the pathogenesis of NeuroAIDS. J Leukoc Biol 79: 444–452. 16507710 10.1189/jlb.0405215 PMC2505195

[15] Kim YC, Bang D, Lee S, Lee KH (2000) The effect of herpesvirus infection on the expression of cell adhesion molecules on cultured human dermal microvascular endothelial cells. J Dermatol Sci 24: 38–47. 10960777 10.1016/s0923-1811(00)00080-3

[16] Roe K, Kumar M, Lum S, Orillo B, Nerurkar VR, et al (2012) West Nile virus-induced disruption of the blood-brain barrier in mice is characterized by the degradation of the junctional complex proteins and increase in multiple matrix metalloproteinases. J Gen Virol 93: 1193–1203. 22398316 10.1099/vir.0.040899-0 PMC3755517

[17] Verma S, Lo Y, Chapagain M, Lum S, Kumar M, et al (2009) West Nile virus infection modulates human brain microvascular endothelial cells tight junction proteins and cell adhesion molecules: Transmigration across the in vitro blood-brain barrier. Virology 385: 425–433. 19135695 10.1016/j.virol.2008.11.047 PMC2684466

[18] Verma S, Kumar M, Gurjav U, Lum S, Nerurkar VR (2010) Reversal of West Nile virus-induced blood-brain barrier disruption and tight junction proteins degradation by matrix metalloproteinases inhibitor. Virology 397: 130–138. 19922973 10.1016/j.virol.2009.10.036 PMC3102050

[19] Shrestha B, Diamond MS (2004) Role of CD8+ T cells in control of West Nile virus infection. J Virol 78: 8312–8321. 15254203 10.1128/JVI.78.15.8312-8321.2004 PMC446114

[20] King NJ, Getts DR, Getts MT, Rana S, Shrestha B, et al (2007) Immunopathology of flavivirus infections. Immunol Cell Biol 85: 33–42. 17146465 10.1038/sj.icb.7100012

[21] Wang T, Welte T (2013) Role of natural killer and Gamma-delta T cells in West Nile virus infection. Viruses 5: 2298–2310. 24061543 10.3390/v5092298 PMC3798903

[22] Lim JK, Obara CJ, Rivollier A, Pletnev AG, Kelsall BL, et al (2011) Chemokine receptor Ccr2 is critical for monocyte accumulation and survival in West Nile virus encephalitis. J Immunol 186: 471–478. 21131425 10.4049/jimmunol.1003003 PMC3402345

[23] Shen J, SS TT, Schrieber L, King NJ (1997) Early E-selectin, VCAM-1, ICAM-1, and late major histocompatibility complex antigen induction on human endothelial cells by flavivirus and comodulation of adhesion molecule expression by immune cytokines. J Virol 71: 9323–9332. 9371591 10.1128/jvi.71.12.9323-9332.1997 PMC230235

[24] Getts DR, Terry RL, Getts MT, Muller M, Rana S, et al (2012) Targeted blockade in lethal West Nile virus encephalitis indicates a crucial role for very late antigen (VLA)-4-dependent recruitment of nitric oxide-producing macrophages. J Neuroinflammation 9: 246. 23111065 10.1186/1742-2094-9-246 PMC3532418

[25] Dai J, Wang P, Bai F, Town T, Fikrig E (2008) Icam-1 participates in the entry of west nile virus into the central nervous system. J Virol 82: 4164–4168. 18256150 10.1128/JVI.02621-07 PMC2292986

[26] van Marle G, Antony J, Ostermann H, Dunham C, Hunt T, et al (2007) West Nile virus-induced neuroinflammation: glial infection and capsid protein-mediated neurovirulence. J Virol 81: 10933–10949. 17670819 10.1128/JVI.02422-06 PMC2045515

[27] Arjona A, Foellmer HG, Town T, Leng L, McDonald C, et al (2007) Abrogation of macrophage migration inhibitory factor decreases West Nile virus lethality by limiting viral neuroinvasion. J Clin Invest 117: 3059–3066. 17909632 10.1172/JCI32218 PMC1994625

[28] Kumar M, Roe K, Orillo B, Muruve DA, Nerurkar VR, et al (2013) Inflammasome adaptor protein Apoptosis-associated speck-like protein containing CARD (ASC) is critical for the immune response and survival in west Nile virus encephalitis. J Virol 87: 3655–3667. 23302887 10.1128/JVI.02667-12 PMC3624239

[29] Bai F, Town T, Qian F, Wang P, Kamanaka M, et al (2009) IL-10 signaling blockade controls murine West Nile virus infection. PLoS Pathog 5: e1000610. 19816558 10.1371/journal.ppat.1000610 PMC2749443

[30] Shrestha B, Zhang B, Purtha WE, Klein RS, Diamond MS (2008) Tumor necrosis factor alpha protects against lethal West Nile virus infection by promoting trafficking of mononuclear leukocytes into the central nervous system. J Virol 82: 8956–8964. 18632856 10.1128/JVI.01118-08 PMC2546880

[31] Dobbie MS, Hurst RD, Klein NJ, Surtees RA (1999) Upregulation of intercellular adhesion molecule-1 expression on human endothelial cells by tumour necrosis factor-alpha in an in vitro model of the blood-brain barrier. Brain Res 830: 330–336. 10366690 10.1016/s0006-8993(99)01436-5

[32] Eugenin EA, Osiecki K, Lopez L, Goldstein H, Calderon TM, et al (2006) CCL2/monocyte chemoattractant protein-1 mediates enhanced transmigration of human immunodeficiency virus (HIV)-infected leukocytes across the blood-brain barrier: a potential mechanism of HIV-CNS invasion and NeuroAIDS. J Neurosci 26: 1098–1106. 16436595 10.1523/JNEUROSCI.3863-05.2006 PMC6674577

[33] Lee YR, Liu MT, Lei HY, Liu CC, Wu JM, et al (2006) MCP-1, a highly expressed chemokine in dengue haemorrhagic fever/dengue shock syndrome patients, may cause permeability change, possibly through reduced tight junctions of vascular endothelium cells. J Gen Virol 87: 3623–3630. 17098977 10.1099/vir.0.82093-0

[34] Lachenmaier SM, Deli MA, Meissner M, Liesenfeld O (2011) Intracellular transport of Toxoplasma gondii through the blood-brain barrier. J Neuroimmunol 232: 119–130. 21106256 10.1016/j.jneuroim.2010.10.029 PMC4116595

[35] Mestre L, Inigo PM, Mecha M, Correa FG, Hernangomez-Herrero M, et al (2011) Anandamide inhibits Theiler's virus induced VCAM-1 in brain endothelial cells and reduces leukocyte transmigration in a model of blood brain barrier by activation of CB(1) receptors. J Neuroinflammation 8: 102. 21851608 10.1186/1742-2094-8-102 PMC3173342

[36] Wu DT, Woodman SE, Weiss JM, McManus CM, D'Aversa TG, et al (2000) Mechanisms of leukocyte trafficking into the CNS. J Neurovirol 6 Suppl 1: S82–85. 10871769

[37] Schafer A, Brooke CB, Whitmore AC, Johnston RE (2011) The Role of the Blood-Brain Barrier during Venezuelan Equine Encephalitis Virus Infection. J Virol 85: 10682–10690. 21849461 10.1128/JVI.05032-11 PMC3187510

[38] Bai F, Kong KF, Dai J, Qian F, Zhang L, et al (2010) A paradoxical role for neutrophils in the pathogenesis of West Nile virus. J Infect Dis 202: 1804–1812. 21050124 10.1086/657416 PMC3053000

[39] Kanwar JR, Kanwar RK, Wang D, Krissansen GW (2000) Prevention of a chronic progressive form of experimental autoimmune encephalomyelitis by an antibody against mucosal addressin cell adhesion molecule-1, given early in the course of disease progression. Immunol Cell Biol 78: 641–645. 11114975 10.1046/j.1440-1711.2000.00947.x

[40] Steiner O, Coisne C, Cecchelli R, Boscacci R, Deutsch U, et al (2010) Differential roles for endothelial ICAM-1, ICAM-2, and VCAM-1 in shear-resistant T cell arrest, polarization, and directed crawling on blood-brain barrier endothelium. J Immunol 185: 4846–4855. 20861356 10.4049/jimmunol.0903732

[41] Beasley DW, Li L, Suderman MT, Barrett AD (2002) Mouse neuroinvasive phenotype of West Nile virus strains varies depending upon virus genotype. Virology 296: 17–23. 12036314 10.1006/viro.2002.1372

[42] Scherbik SV, Stockman BM, Brinton MA (2007) Differential expression of interferon (IFN) regulatory factors and IFN-stimulated genes at early times after West Nile virus infection of mouse embryo fibroblasts. J Virol 81: 12005–12018. 17804507 10.1128/JVI.01359-07 PMC2168811

[43] Hussmann KL, Samuel MA, Kim KS, Diamond MS, Fredericksen BL (2013) Differential replication of pathogenic and nonpathogenic strains of West Nile virus within astrocytes. J Virol 87: 2814–2822. 23269784 10.1128/JVI.02577-12 PMC3571364

[44] Hussmann KL, Fredericksen BL (2014) Differential induction of CCL5 by pathogenic and nonpathogenic strains of West Nile virus in brain endothelial cells and astrocytes. J Gen Virol. 10.1099/vir.0.060558-0 PMC3973477 24413421

[45] Hasebe R, Suzuki T, Makino Y, Igarashi M, Yamanouchi S, et al (2010) Transcellular transport of West Nile virus-like particles across human endothelial cells depends on residues 156 and 159 of envelope protein. BMC Microbiol 10: 165. 20529314 10.1186/1471-2180-10-165 PMC2889955

[46] Greenwood J, Heasman SJ, Alvarez JI, Prat A, Lyck R, et al (2011) Review: leucocyte-endothelial cell crosstalk at the blood-brain barrier: a prerequisite for successful immune cell entry to the brain. Neuropathol Appl Neurobiol 37: 24–39. 20946472 10.1111/j.1365-2990.2010.01140.x

[47] Giri R, Selvaraj S, Miller CA, Hofman F, Yan SD, et al (2002) Effect of endothelial cell polarity on beta-amyloid-induced migration of monocytes across normal and AD endothelium. Am J Physiol Cell Physiol 283: C895–904. 12176746 10.1152/ajpcell.00293.2001

[48] Verma S, Molina Y, Lo YY, Cropp B, Nakano C, et al (2008) In vitro effects of selenium deficiency on West Nile virus replication and cytopathogenicity. Virol J 5: 66. 18513435 10.1186/1743-422X-5-66 PMC2453119

[49] Wong D, Prameya R, Dorovini-Zis K (2007) Adhesion and migration of polymorphonuclear leukocytes across human brain microvessel endothelial cells are differentially regulated by endothelial cell adhesion molecules and modulate monolayer permeability. J Neuroimmunol 184: 136–148. 17291598 10.1016/j.jneuroim.2006.12.003

